# Expression of Partitioning Defective 3 (Par-3) for Predicting Extrahepatic Metastasis and Survival with Hepatocellular Carcinoma

**DOI:** 10.3390/ijms14011684

**Published:** 2013-01-15

**Authors:** Yee-Jee Jan, Bor-Sheng Ko, Tzu-An Liu, Yao-Ming Wu, Shu-Man Liang, Shyh-Chang Chen, John Wang, Jun-Yang Liou

**Affiliations:** 1 Department of Pathology and Laboratory Medicine, Taichung Veterans General Hospital, Taichung 407, Taiwan; E-Mails: yejan@vghtc.gov.tw (Y.-J.J.); jesica2700@yahoo.com.tw (S.-C.C.); shengw@seed.net.tw (J.W.); 2 Institute of Cellular and System Medicine, National Health Research Institutes, Zhunan 350, Taiwan; E-Mails: kevinkomd@gmail.com (B.-S.K.); ann.liu@nhri.org.tw (T.-A.L.); shu-man@nhri.org.tw (S.-M.L.); 3 Department of Internal Medicine, National Taiwan University Hospital, Taipei 100, Taiwan; 4 Department of Surgery, National Taiwan University Hospital, Taipei 100, Taiwan; E-Mail: wyaoming@gmail.com; 5 Graduate Institute of Basic Medical Science, China Medical University, Taichung 404, Taiwan

**Keywords:** hepatocellular carcinoma, metastasis, Par-3, survival

## Abstract

Partitioning defective 3 (Par-3), a crucial component of partitioning-defective complex proteins, controls cell polarity and contributes to cell migration and cancer cell epithelial-to-mesenchymal transition. However, the clinical relevance of Par-3 in tumor progression and metastasis has not been well elucidated. In this study, we investigated the impact and association of Par-3 expression and clinical outcomes with hepatocellular carcinoma (HCC). We first confirmed that Par-3 was abundantly expressed in HCC cell lines by Western blot analysis. We used immunohistochemistry to analyze the association of Par-3 expression and clinicopathological characteristics in primary and subsequent metastatic tumors of patients with HCC. Par-3 was overexpressed in 47 of 111 (42.3%) primary tumors. Increased expression of Par-3 in primary tumors predicted an increased five-year cumulative incidence of extrahepatic metastasis. In addition, multivariate analysis revealed that Par-3 overexpression was an independent risk factor of extrahepatic metastasis. Increased Par-3 expression in primary tumors was associated with poor five-year overall survival rates and was an independent prognostic factor on Cox regression analysis. In conclusion, we show for the first time that increased Par-3 expression is associated with distant metastasis and poor survival rates in patients with HCC. Par-3 may be a novel prognostic biomarker and therapeutic target for HCC.

## 1. Introduction

Hepatocellular carcinoma (HCC) is a serious malignancy and public health problem in endemic areas of hepatitis B or C virus infection, including Africa and Southeast Asia [1]. Despite the aggressive surgical and non-surgical approaches used to treat and improve the outcome of HCC [2], local recurrence and distant metastasis remain major causes of treatment failure [3,4]. Investigating accurate prognostic biomarkers for early detection and prediction of recurrence and metastasis is critical for developing novel therapeutic strategies to improve outcome and survival for HCC patients.

Cell polarity is a fundamental property of all eukaryotic cells and is essential for the cell development of various organisms. Dysfunction of polarity leads to distinct diseases, including cancer progression [5]. The partitioning defective (Par) complex comprises several proteins, including Par-3, Par-6 and atypical protein kinase C (aPKC), which regulate cell polarity and migration by regulating protein-protein interaction with several GTP-bound regulators [6–8]. In mammalian epithelial cells, the Par complex localizes to the apical junction region and plays a critical role in establishing apical-basal polarity and tight junctions [9–12]. Thus, the dynamic balance and regulation of the polarity-related proteins containing Par complex members are extremely important to modulate cancer cell migration and epithelial-to-mesenchymal transition. Dissolution of cell-cell junctions with loss of

Par-3 or Par-6 expression promotes cancer cell migration and invasion [8,13]. Conversely, amplification and increased expression of Par-6 and aPKC induced cell proliferation, more aggressive tumors and poor outcomes in breast cancer [14], ovarian cancer [15] and non-small-cell lung cancer [16]. Par-3 expression and regulation are considered largely involved in cancer cell migration, and a few studies have suggested defective expression or amplified *PARD3* gene in prostate cancer cells [17], esophageal squamous cell carcinoma [18], neoplastic retinal pigment epithelial cells [19] and HCC [20]. Thus, Par-3 may play an important role in tumor development and cancer cell progression. However, the clinical significance of Par-3 expression in tumor metastasis and survival has never been elucidated. Therefore, in this study, we investigated Par-3 expression by immunohistochemistry in a cohort of patients with HCC. We evaluate the association of Par-3 expression with clinicopathological characteristics and survival rates. Par-3 overexpression was significantly associated with extrahepatic metastasis in HCC, and increased Par-3 expression was associated with worse overall survival with HCC. Our results suggest Par-3 as a potential biomarker and therapeutic target of HCC.

## 2. Results

### 2.1. Protein Expression of Par-3 in HCC Cell Lines

**Figure 1:**
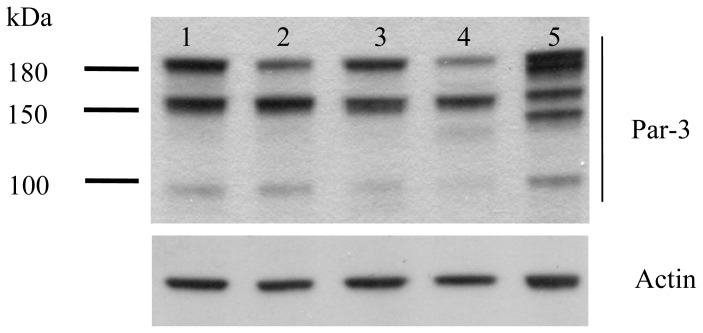
Protein expression of Partitioning defective 3 (Par-3) in hepatocellular carcinoma (HCC) cell lines determined by Western blot analysis. Lane 1, Huh-7; lane 2, HepG2; lane 3, Hep3B; lane 4, PLC-5; lane 5, SK-Hep-1 cells.

Western blot analysis revealed Par-3 protein variants of 180, 150 and 100 kDa with differential expression in all HCC cell lines (Huh-7, HepG2, Hep3B, PLC-5 and SK-Hep-1) (Figure 1). Interestingly, the poorly-differentiated HCC cells, SK-Hep-1, expressed more multiple forms of Par-3 protein variants than other well-differentiated HCC cell lines.

### 2.2. Increased Par-3 Protein Expression in Primary and Metastatic HCC Tissues and Association with HCC Extrahepatic Metastasis

**Figure 2:**
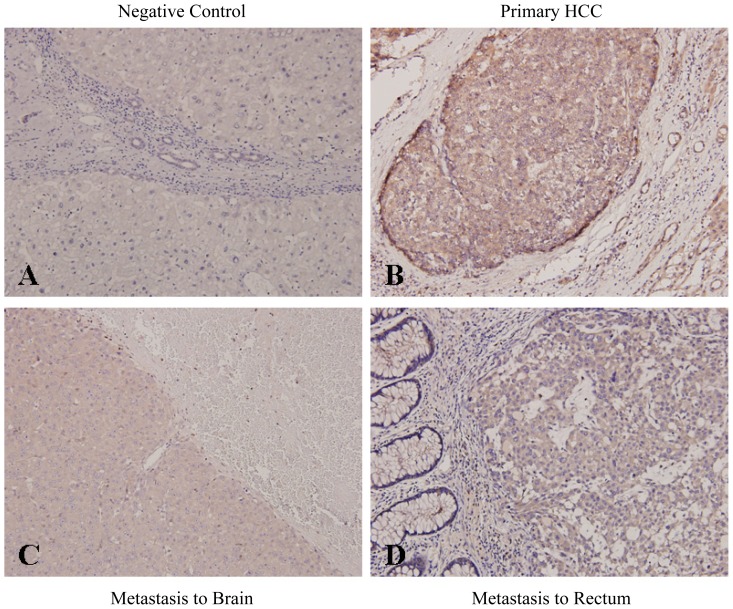
Immunohistochemical analysis of Par-3 in primary and metastatic HCC tissues. (**A**) Negative control staining (200×); (**B**) Par-3 staining in representative primary HCC (200×); Staining of Par-3 in representative metastatic HCC lesions in (**C**) brain and (**D**) rectum (200×). Negative Control Primary HCC Metastasis to Brain Metastasis to Rectum

We examined the expression of Par-3 in paraffin-embedded primary HCC tumors with surrounding non-cancerous parenchyma from 111 patients and 31 matched extrahepatic metastatic tumors by immunohistochemical staining. Negative control slides were negatively unstained with Par-3 (Figure 2A). The expression of Par-3 was increased in 47 (42.3%) of 111 primary HCC tumors and not in non-cancerous cells adjacent to tumors (Figure 2B and Table 1). Moreover, Par-3 was overexpressed in 31 matched metastatic HCC specimens, as illustrated in brain (Figure 2C) and rectum (Figure 2D). Expression of Par-3 was not significantly related to most clinicopathological characteristics, but was associated with tumor multiplicity (*p* = 0.002), Alpha-fetoprotein level (*p* = 0.046) and subsequent extrahepatic metastasis (*p* = 0.037) (Table 1).

**Figure 3:**
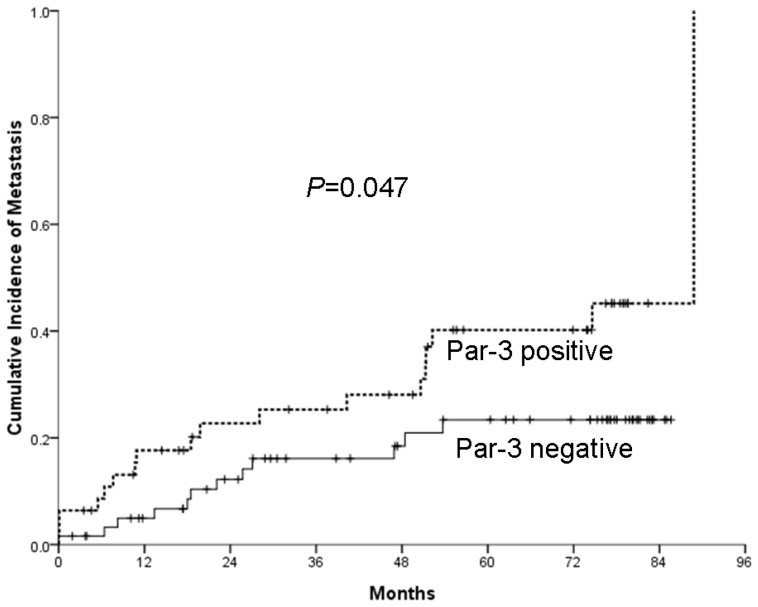
Par-3 positivity is associated with risk of HCC metastasis. Five-year cumulative risk of extrahepatic metastasis with HCC with positive and negative Par-3 (40.2% ± 8.0% *vs.* 23.4% ± 6.0%, *p =* 0.047).

Multivariate analysis confirmed Par-3 expression as a predictor of distant HCC metastasis (*p =* 0.037) (Table 2). The cumulative rate of developing extrahepatic metastasis within five years with primary HCC was significantly higher with positive rather than negative Par-3 expression (40.2% ± 8.0% *vs.* 23.4% ± 6.0%, *p =* 0.047) (Figure 3). Furthermore, the expression of Par-3 was significantly increased in metastatic HCC samples than in their primary tumors (21 with increased Q-score > 2, and 10 with no difference in Q-score, *p* < 0.001). These observations suggest a strong association of Par-3 expression and extrahepatic metastasis of HCC.

### 2.3. Overexpression of Par-3 and HCC Patient Survival

**Figure 4:**
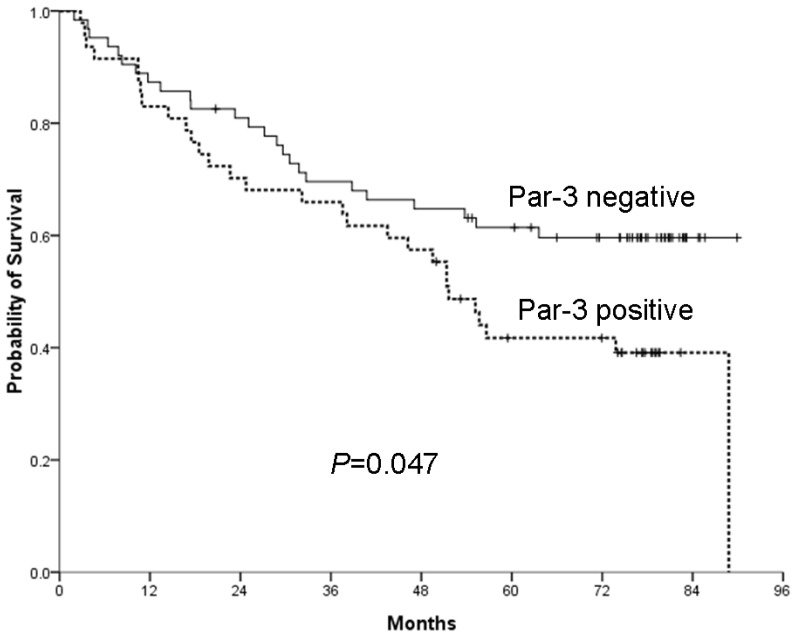
Par-3 positivity is associated with poor five-year survival with HCC. Five-year cumulative survival with HCC with positive and negative Par-3 (41.7% ± 7.3% *vs.* 59.6% ± 6.3%, *p =* 0.047).

After a mean follow-up of 52.0 ± 28.4 months after surgery, 27 patients (24.3%) remained free of HCC, 54 patients (48.6%) had died because of their disease and 30 patients (27.0%) were still alive with disease recurrence and/or distant metastasis. Survival analysis revealed a significantly better overall five-year survival with negative rather than positive Par-3 expression in primary HCC tumors (59.6% ± 6.3% *vs.* 41.7% ± 7.3%, *p =* 0.047) (Figure 4). The increased expression of Par-3 in primary tumors had no significant effect on progression-free survival in these patients (data not shown). In addition, Cox proportional-hazard regression models revealed that Par-3 overexpression was significantly associated with poor overall survival (hazard ratio 2.049, 95% confidence interval 1.082–3.884, *p =* 0.028), but not associated with progression-free survival (Table 3). Thus, overexpression of Par-3 in primary tumors is an important predictor of poor overall survival with HCC.

### 2.4. Correlation of Par-3 Expression with 14-3-3ɛ

**Figure 5:**
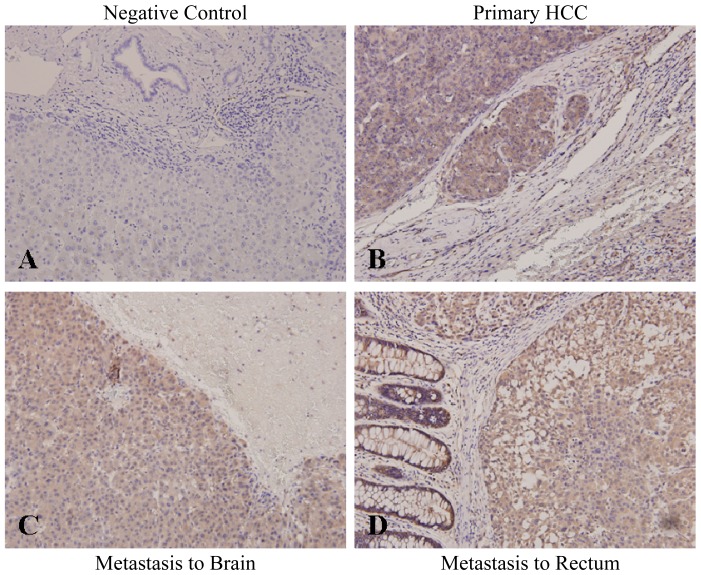
Immunohistochemical analysis of 14-3-3ɛ in primary and metastatic HCC tissues. (**A**) Negative control staining (200×); (**B**) 14-3-3ɛ staining in representative primary HCC (200×); Staining of 14-3-3ɛ in representative metastatic HCC lesions in (**C**) brain and (**D**) rectum (200×).

To examine whether Par-3 expression is associated with 14-3-3ɛ (Par-5), we determined the 14-3-3ɛ expression by IHC analysis. Negative control slides were unstained with 14-3-3ɛ (Figure 5A). 14-3-3ɛ was significantly overexpressed in primary (Figure 5B) and metastatic HCC tumors, as representatively illustrated in brain and rectum (Figure 5C,D). Furthermore, overexpression of 14-3-3ɛ is significantly correlated with Par-3 in primary HCC tumors (*p =* 0.014) (Table 1).

## 3. Discussion

Cell polarity is a basic and fundamental property of regular multiple cellular functions, including cancer cell migration and epithelial-to-mesenchymal transition. The Par-3/Par-6/aPKC complex is an essential regulator controlling cell polarity via interacting with various proteins. Human Par-3 (*PARD3*) is a single-copy gene consisting of 26 exons and localized in chromosome 10 [20]. At least five *PARD3* variants, derived from alternative splicing and polyadenylation, have been identified in a human liver cDNA library [20]. Furthermore, multiple-splice *PARD3* gene variants [21–23] and variants with three main molecular weights (180, 150 and 100 kDa) have been reported [18,24], although their specific role remains unclear. However, Par-3 expression in some tumors has been controversial. For instance, Par-3 protein or RNA expression was downregulated in esophageal squamous cell carcinoma and HCC [18,20], but *PARD3* gene was found mutationally inactivated in prostate cancer cells [17]. In contrast, gene amplification of aPKC-binding Par-3 protein was reported in transformed neoplastic retinal pigment epithelial cells [19]. Par-3 was reported to localize and regulate epithelial tight junction assembly, which was promoted by epidermal growth factor receptor (EGFR) [24] and TGF-β [25] signaling. Moreover, overexpression of Par-3 suppresses contact-mediated inhibition of cell migration [26]. Thus, Par-3 may be a “double-edged sword” in regulating cell migration and epithelial-mesenchymal transition (EMT), depending on the cell type or tissue. Also, the diverse role of Par-3 may be attributed to the distinct variants with different molecular weights, which may explain the Par-3 protein expression in the most migratory and poorly differentiated HCC cell line, SK-Hep-1, differing from that of the other cell lines (Figure 1). Nevertheless, little is known about whether and how Par-3 variants regulate cell polarity and contribute to cancer cell migration or EMT.

14-3-3 (also known as Par-5) potentially modulates cell polarity by directly binding with phosphorylated Par-3 [9,12,27]. We previously reported the expression of 14-3-3β, 14-3-3γ and 14-3-3ɛ isoforms increased in HCC, and their overexpression predicted a poor outcome with HCC [28–30]. Interestingly, we noted a significant association of Par-3 expression and 14-3-3, particularly 14-3-3ɛ, in primary and metastatic HCC (Figure 2*vs.*Figure 5, Table 1). Our immunoprecipitation findings also revealed that Par-3 directly interacts with 14-3-3ɛ to form a complex in HCC cells (unpublished data). Although the detailed mechanism of association or interaction between 14-3-3 proteins and Par-3 in tumor progression has not been elucidated, our results suggest that Par-3 may interact and collaborate with 14-3-3 to synergize HCC tumor progression. Further work is ongoing to uncover the role of Par-3/Par-6/aPKC complex interacting with 14-3-3 in regulating HCC development.

Results from this study indicate that increased Par-3 expression participates in promoting distant metastasis and reducing the survival rate of HCC patients. Elevated Par-3 expression in primary tumors is associated with risk of extrahepatic metastasis and poor overall survival with HCC. Thus, Par-3 alone or in combination with 14-3-3 proteins may be a biological marker identifying HCC patients at high risk of metastasis and poor survival. Therapeutic strategies or drugs aimed at Par-3 or 14-3-3 proteins might be developed for these patients.

## 4. Materials and Methods

### 4.1. Patients and Clinical Specimens

We retrospectively enrolled (from January 1999 to December 2001) and obtained tissue from 111 HCC patients who underwent surgery for tumor resection in Taichung Veterans General Hospital. The mean follow-up was 52.0 ± 28.4 months. In total, tissue from 31 patients (27.9%) showed metastasis 5 to 88 months after the surgery for primary HCC. The metastasis sites included bone, abdominal and chest wall, brain, mesentery, peritoneum, adrenal gland and retroperitoneum. The paraffin-embedded surgical specimens composed of the primary tumors with surrounding non-cancerous liver parenchyma and metastatic tumors underwent pathology examination. We examined pathological features, including Barcelona-Clinic Liver Cancer (BCLC) [31] staging and clinical outcomes. This study was approved by the Institutional Review Board of Taichung Veterans General Hospital.

### 4.2. Immunohistochemical Analysis

For immunohistochemistry analysis of Par-3 expression in paraffin-embedded tissues, we used an automatic immunostaining device and ultraView detection kit (Ventana XT Medical System, Tucson, AZ, USA) with a primary rabbit polyclonal antibody for Par-3 (1:100, Millipore, Clone 07–330, Temecula, CA, USA). Detection of 14-3-3ɛ expression was performed according to [28], described previously. A negative control was incubation without the primary antibody. The intensity of Par-3 and 14-3-3ɛ proteins staining were semiquantitatively scored by a Quick-score (Q-score) method based on intensity and heterogeneity [28–30,32–35]. Staining intensity was scored as 0 (negative), 1 (weak), 2 (moderate) or 3 (strong). For heterogeneity, the proportion of tumor cells positively stained with Par-3 was scored as 0 (0%), 1 (1%–25%), 2 (26%–50%), 3 (51%–75%) and 4 (76%–100%). The Q-score of a given tissue sample was the sum of intensity and heterogeneity scores and ranged from 0 to 7. The scoring of each sample was evaluated independently and blindly by 2 pathologists. A Q-score ≥2 was considered to be an overexpressed or positive expression of Par-3/14-3-3ɛ, and a Q-score <2 was considered a normal or negative expression of Par-3/14-3-3ɛ. Some rare cases with <5% weakly stained specimens were considered to be a negative expression.

### 4.3. Cell Culture and Western Blot Analysis

Huh-7, HepG2, Hep3B, PLC-5 and SK-Hep-1 human hepatoma cells were maintained in DMEM (Gibco, Gaithersburg, MD, USA), supplemented with 10% fetal bovine serum (FBS; Hyclone Thermo Fisher Scientific, Waltham, MA, USA), 100 units/mL penicillin and 100 units/mL streptomycin in a humidified incubator with 5% CO_2_ at 37 °C. Expression of Par-3 protein was determined by Western blot analysis. In brief, HCC cells were cultured to 90% confluence, harvested and lysed in ice-cold RIPA buffer (0.5 M Tris-HCl, pH 7.4, 1.5 M NaCl, 2.5% deoxycholic acid, 10% NP-40, 10 mM EDTA; Millipore, Temecula, CA, USA) containing cocktail protease inhibitors (Roche, Indianapolis, IN, USA). Cell lysates were clarified by centrifugation at 15,000 rpm for 20 min at 4 °C. Protein concentration was determined by use of a Bio-Rad protein assay kit (Bio-Rad Laboratories, Hercules, CA, USA). In total, 20 μg protein from each sample was applied to a gradient SDS-PAGE gel and immunoblotted onto PVDF membranes, which were blocked for 1 h in PBST (0.1% Tween 20, 2.67 mM KCl, 1.47 mM KH_2_PO_4_, 137.93 mM NaCl, 8.1 mM Na_2_HPO_4_, pH 7.4) containing 5% nonfat dry milk, then incubated with primary antibody against Par-3 (Sigma-Aldrich, St. Louis, MO, USA) overnight, washed 3 times with PBST for 5 min, then incubated with horseradish peroxidase-conjugated secondary antibody for 1 h. Protein levels were determined by use of enhanced chemiluminescence reagents.

### 4.4. Statistical Analysis

One-Way ANOVA was used to analyze differences for clinicopathological variables. Multivariate logistic regression was used to determine factors predicting extrahepatic metastasis. The Wilcoxon signed-rank test was used to analyze the differences between primary tumors and matched metastatic tissues by Par-3 staining density. Kaplan-Meier curves were plotted, and the log rank test was used to analyze time-related probabilities of metastasis, overall survival and progression-free survival. Cox proportional hazards regression models were used to evaluate the impact of prognostic factors on survival. *p* < 0.05 was considered statistically significant and *p =* 0.05 to 0.10 marginally significant.

## 5. Conclusions

In this study, we show for the first time that expression of Par-3 is increased and significantly associated with poor prognostic outcomes of HCC patients. To further investigate the molecular mechanism by which Par-3 is involved in regulating HCC tumors will benefit the implication of diagnosis or treatment for HCC. Thus, Par-3 alone or combined with 14-3-3ɛ or related interacting components may serve as the potential markers or therapeutic targets of HCC.

## Figures and Tables

**Table 1:** Correlation between Par-3 expression in primary tumor and clinicopathological characteristics of hepatocellular carcinoma patients.

Parameters	Par-3 positivity (Q-score ≥ 2)% (*n*)	*p*-Value
Overall (*n* = 111)	42.3% (47)	
Age		
≥60 years (*n =* 55)	40.0% (22)	NS
<60 years (*n =* 56)	44.6% (25)	
Gender		
Male (*n =* 84)	42.9% (36)	NS
Female (*n =* 27)	40.7% (11)	
Histology grade		
1 (*n =* 7)	42.9% (3)	NS
2 (*n =* 79)	41.8% (33)	
3 (*n =* 25)	44.0% (11)	
Types of surgery		
Wedge resection (*n =* 39)	38.5% (15)	NS
Segmentectomy (*n =* 54)	38.9% (21)	
Lobectomy (*n =* 18)	61.1% (11)	
Surgical margin		
Free (*n =* 84)	42.9% (36)	NS
Involved (*n =* 27)	40.7% (11)	
BCLC staging		
Not available (*n =* 5)		
Early (stage A1 to A4) (*n =* 56)	44.6% (25)	NS
Intermediate (stage B) (*n =* 49)	40.8% (20)	
Advanced (stage C) (*n =* 1)	100.0% (1)	
Tumor size		
≥5.0 cm (*n =* 36)	50.0% (18)	NS
<5.0 cm (*n =* 75)	38.7% (29)	
Tumor multiplicity		
Single (*n =* 86)	50.0% (43)	0.002 *
Multiple (*n =* 25)	16.0% (4)	
Capsular formation		
Not available (*n =* 8)		NS
Yes (*n =* 60)	41.7% (25)	
No (*n =* 43)	39.5% (17)	
Micro-vascular thrombi		
Yes (*n =* 48)	45.8% (22)	NS
No (*n =* 63)	39.7% (25)	
Liver cirrhosis		
Not available (*n =* 3)		NS
Yes (*n =* 56)	48.2% (27)	
No (*n =* 52)	38.5% (20)	
Viral hepatitis		
Not available (*n =* 7)		
Hepatitis B (*n =* 56)	41.1% (23)	NS
Hepatitis C (*n =* 30)	43.3% (13)	
Both (*n =* 15)	40.0% (6)	
None (*n =* 3)	66.7% (2)	
Alpha-fetoprotein level		
Not available (*n =* 12)		0.046 *
≥80 ng/mL (*n =* 36)	55.6% (20)	
<80 ng/mL (*n =* 63)	34.9% (22)	
Subsequent extrahepatic metastasis		
Yes (*n =* 31)	58.1% (18)	0.037 *
No (*n =* 80)	36.3% (29)	
14-3-3ɛ expression		
Yes (*n =* 68)	51.5% (35)	0.014 *
No (*n =* 43)	27.9% (12)	

BCLC, Barcelona-Clinic Liver Cancer; NS, not significant; Q-score, Quick-score; SD, standard deviation;

* *p* < 0.05.

**Table 2:** Multivariate analysis for distant metastasis in hepatocellular carcinoma patients.

Variables	*p*-Value
Histology grade (1 + 2 : 3)	NS
Presence of liver cirrhosis (No : Yes)	NS
Par-3 expression (negative : positive)	0.037 *
Bulky tumor (≥5.0 cm : <5.0 cm)	NS
Surgical margin (free : involved)	NS
Capsule formation (no : yes)	NS
Vascular thrombi (no : yes)	NS
BCLC staging (Stage A : B to C)	NS

BCLC, Barcelona-Clinic Liver Cancer; NS, not significant.

* *p* < 0.05.

**Table 3:** Cox proportional hazard regression analysis for survival in hepatocellular carcinoma patients.

Variables	Overall survival		Progression-free survival	
	
	Hazard ratio (95% CI)	*p*-Value	Hazard ratio (95% CI)	*p*-Value
Par-3 expression (negative : positive)	2.049 (1.082–3.884)	0.028 *	1.486 (0.886–2.490)	0.133

Types of operation (wedge : wider resection)	0.719 (0.269–1.922)	0.510	0.756 (0.431–1.325)	0.329

Surgical margin (free : involved)	1.510 (0.763–2.988)	0.237	1.061 (0.580–1.941)	0.847

Capsular formation (no : yes)	1.357 (0.723–2.547)	0.342	1.543 (0.915–2.602)	0.104

Alpha-fetoprotein level (low : high)	1.824 (0.986–3.412)	0.059 #	1.757 (1.064–2.898)	0.028 *

Liver cirrhosis (no : yes)	1.294 (0.651–2.571)	0.463	1.059 (0.598–1.875)	0.845

BCLC stage (A : B and C)	1.861 (0.902–3.838)	0.093 #	1.555 (0.872–2.775)	0.135

CI, confidence interval; BCLC, Barcelona-Clinic Liver Cancer; SE, standard error;

* *p* < 0.05;

# 0.05 < *p* < 0.10.
